# New approaches to explanting high-viscosity silicone oil in retinal surgery—polyimide cannulas vs extraction sleeves vs a luer-trocar

**DOI:** 10.1186/s40942-023-00481-w

**Published:** 2023-07-24

**Authors:** Maximilian Hammer, Sonja Schickhardt, Donald Munro, Alexander Scheuerle, Ramin Khoramnia, Philipp Uhl, Gerd U. Auffarth

**Affiliations:** 1 David J Apple Laboratory for Vision Research, Heidelberg, Germany; 2 University Eye Hospital Heidelberg, Im Neuenheimer Feld 400, 69120 Heidelberg, Germany; 3 Institute for Pharmacology and Molecular Biotechnology, University of Heidelberg, Heidelberg, Germany

**Keywords:** Vitreoretinal surgery, Retinal surgery, Silicone oil, Endotamponade, removal, Cannula, Surgical technique

## Abstract

**Background:**

The removal of high-viscosity silicone oil in retinal surgery is time-intensive. In this laboratory and porcine eye study, we evaluated the efficiency of novel extraction techniques, namely shortened polyimide-cannulas, an extraction sleeve and an experimental luer-trocar system, for commercially available silicone oils.

**Methods:**

The volume of silicone oil after a 5-min removal time was quantified. The feasibility of all studied techniques was additionally tested in vitrectomized porcine eyes. Siluron 1000, 2000, 5000 and Xtra, as well as Densiron 68 and Densiron Xtra were examined.

**Results:**

Shortening the 23G-polyimide-cannula from 6 mm up to 1.5 mm led to a significantly higher mean aspirated volume (e.g., 1 ml/min to 1.7 ml/min for Siluron Xtra). The extraction sleeve and luer-trocar increased the flow threefold compared to the shortest polyimide cannulas at 23G (5.2 ml/min and 5.2 ml/min vs 1.7 ml/min for Siluron Xtra, respectively). The extraction sleeve and luer-trocar system reached a similar flow at 27G as the best performing 23G-cannula. Angulation and movement of the eye was possible with the 2 mm-cannula, no angulation was possible when using the extraction sleeve. Stability and control were not impaired with the experimental luer-trocar setup.

**Conclusion:**

Shorter polyimide-cannulas and extraction sleeves increase the extraction flow but may decrease stability during surgery. The luer-trocar system facilitates rapid removal while offering great stability and control. Small 27G silicone oil removal is possible and time efficient.

## Background

Silicone oils are used as intraocular endotamponades separately by Amaly and Cibis in 1962 [1, 2] and have become a widespread and accepted long-term option for replacing the vitreous body after vitrectomy for retinal detachment, extensive diabetic retinopathy or endophthalmitis. While in human studies similar anatomic and visual outcomes were achieved with low- and high-viscosity silicone oils [3], high-viscosity silicone oils with viscosities of 5000mPas and above showed lower tendencies to emulsify in in-vitro studies [4–8]. Thus, many vitreoretinal surgeons tend to use high-viscosity silicone oils in most cases.

During surgery, a volume of 4–8 ml of oil is usually injected into and removed from the vitreous cavity after 3 to 12 months. Even at injection pressures of 6 bar this leads to injection times of more than 15 min in case of oils with added high molecular weight components [9, 10]. During silicone oil removal most commonly lower pressures between 600 and 700 mmHg are used further aggravating the surgical downside of these oils. Additionally, considering the upfront trend towards smaller gauge-surgery, new solutions to effectively remove silicone oil from the vitreous cavity are needed [11, 12]. Further, time-efficient techniques may not only translate to time savings during surgery but could also lighten the financial burden on hospitals.

**Fig. 1:**
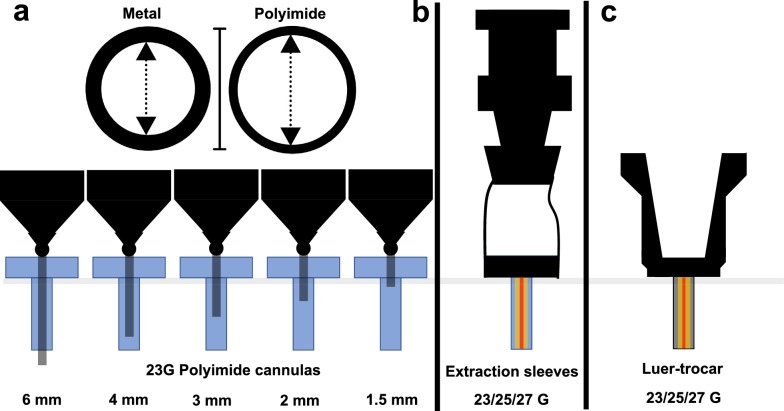
Applied silicone oil removal techniques. **a**: Polyimide cannulas have a thinner wall due to the rigid high-performance polymer polyimide thus increasing the inner diameter. We tested 5 different lengths of the otherwise unchanged 23G-polyimide-cannula in a laboratory study and a porcine eye study. **b**: The extraction sleeve is placed over the trocar, thus using the inner lumen of the trocar for the silicone oil removal process. The use with 23G-, 25G- and 27G-trocars (schematically shown in blue, yellow and red, respectively) was evaluated. **c**: The luer-trocar is a system consisting of a female luer-lock piece attached to a trocar. As such, a stable connection to the syringe is formed. The trocar is used as the lumen. 23G-,25G- and 27G-luer trocar systems (schematically shown in blue, yellow and red, respectively) were used

The laminar flow through a tube can be estimated using the Hagen-Poiseuille equation. The following parameters determine the volumetric flow rate: The pressure difference between the two ends of the tube, the dynamic viscosity of the fluid, the length of the tube and, most importantly, the inner radius/ diameter of the tube. While certain factors, like the viscosity and the pressure difference, have been previously optimized or cannot be optimized, two factors may be improved during surgery, namely the length of the cannula as well as, most importantly, the inner diameter of the cannula. Previously, our group introduced polyimide-cannulas to the scientific literature on vitreoretinal surgery, as they can reduce injection times down to a quarter compared to other commercially available metal cannulas [10]. Polyimide is a rigid, high-performance polymer. The rigidness of the material allows for a slimmer wall thickness, increasing the inner diameter of the cannula while maintaining the same outer diameter. Figure 1 shows the removal techniques applied in this study.

We sought to evaluate if shortening the polyimide-cannula leads to a clinically relevant decrease of removal time. Additionally, another extraction technique are extraction sleeves that are placed over the trocar, thus effectively using the trocar as the lumen. Finally, we tested a prototype of a luer-trocar system: A female luer-lock part is directly attached to the trocar, allowing the direct connection of a syringe to the trocar, thus combining the benefits of cannulas (high control) with using the trocar as the removal lumen (high flow).

## Materials and methods

### Materials

Removal speed was evaluated for six commercially available silicone oils: Siluron 1000, Siluron 2000, Siluron Xtra, Siluron 5000, Densiron 68 and Densiron Xtra (all provided by Fluoron GmbH, Ulm, Germany). The compounds of the tested silicone oils are presented in Table 1. Throughout all experiments, 23G-, 25G- and 27G-trocars were used in conjunction with the removal technique to be tested.

**Table 1:** Compounds and density of examined silicone oils

Name	Compounds	Density at 25 °C
Siluron 1000	100% Polydimethylsiloxane (1000 mPas)	0.97 g/cm3
Siluron 5000	100% Polydimethylsiloxane (5000 mPas)	0.97 g/cm3
Siluron 2000	95% Polydimethylsiloxane (1000 mPas), 5% HMWC (2.5 mio mPas)	0.97 g/cm3
Siluron Xtra	90% Polydimethylsiloxane (1000 mPas), 10% HMWC (2.5 mio mPas)	0.97 g/cm3
Densiron 68	30.5% F6H8, 69.5% Polydimethylsiloxane	1.06 g/cm3
Densiron Xtra	30.5% F6H8, 69.5% Polydimethylsiloxane	1.06 g/cm3

mPas (Millipascal), HMWC (high molecular weight chains), F6H8 (a semi fluorinated alkane**)**

### Cannulas and extraction sleeves

The removal speed was evaluated for 23G-polyimide cannulas (provided by GEUDER AG, Heidelberg, Germany) in different lengths: 6 mm (standard length), 4 mm, 3 mm, 2 mm and 1.5 mm. Results were compared to the removal speed of an extraction sleeve (Alcon, Fort Worth, Texas, USA), which is placed over the trocar after removing the plastic valve. The sleeve was tested in combination with a 23G-, 25G- and a 27G-trocar (provided by GEUDER AG, Heidelberg, Germany). Finally, the prototype of a luer-trocar system was used. It consisted of a standard trocar (23G, 25G, 27G) connected to a female luer-lock part. Figure 1 depicts the different approaches to silicone oil removal tested in this study. A 10 ml syringe was connected to the extraction approaches mentioned above. The syringe was placed in a reusable silicone oil removal kit (Geuder AG, Heidelberg, Germany) connected to a Geuder Megatron S3 (Geuder AG, Heidelberg, Germany).

## Methods

### Experimental setup

A Megatron S3 (GEUDER AG, Heidelberg, Germany) was connected to a reusable silicone oil holder. Using the viscous fluid extraction mode, the vacuum was set to the device maximum of 600 mmHg. The vacuum pressure was constantly monitored throughout all experiments. A trocar was placed in a piece of porcine sclera to mimic the setting in the operating room. The respective cannula was inserted into the trocar, the extraction sleeve was placed on top of the trocar, or the luer-trocar system was placed directly in the sclera. Next, the vacuum was applied for 5 min. The total volume of silicone oil aspirated during these 5 min was quantified. Experiments were carried out threefold for every cannula length, for the extraction sleeve and the luer-trocar for each silicone oil, respectively. The experiments were carried out with the 5-min time limit to ensure that the maximum removal speed while the instrument tip is immersed in the silicone oil bubble is recorded. If 10 ml of an oil sample were aspirated in less than 5 min, the theoretical volume that could be aspirated in 5 min was extrapolated.

### Porcine eye model

To assess if all removal techniques are clinically applicable, we conducted feasibility experiments in vitrectomized, silicone oil-filled pig eyes. The stability and the ability to use the extraction technique to angulate the trocar were evaluated. In this porcine eye model, silicone oil removal was fully completed. A second trocar with a balanced salt solution infusion line was placed and set to a pressure of 40 mmHg to ensure stable intraocular pressure.

### Statistical analysis

To investigate the impact of the shortening of the polyimide-cannula, linear regression analyses were performed for each individual type of silicone oil. To determine the effect of the extraction sleeve and the experimental luer-trocar system on removal time, t-tests between the mean removal time of the shortest cannula and the setup to be tested were performed. Statistical analyses were performed using STATA17BE (StataCorp, College Station, Texas, USA) and Prism 8 (GraphPad Software Inc., San Diego, California, USA).

## Results

### The effect of the cannula length on removal speed

**Fig. 2:**
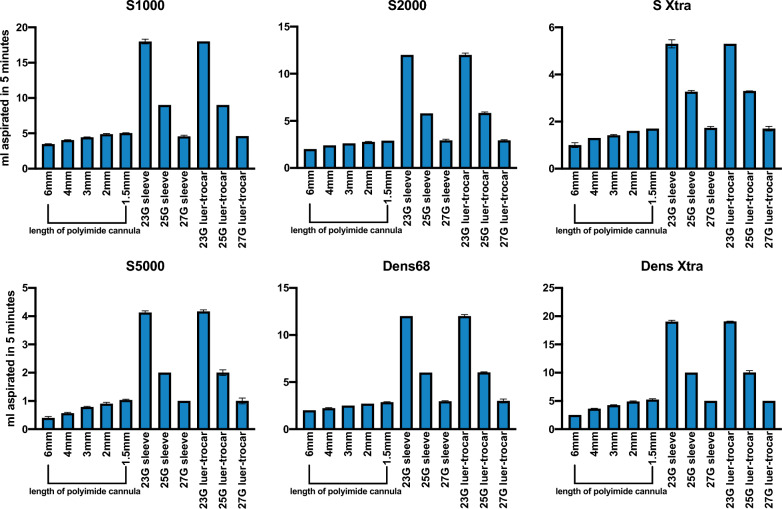
Mean extracted volume in 5 min using different techniques for a variety of clinically used silicone oils. Figure 2 depicts the volume aspirated in 5 min for all tested extraction techniques. Shortening the cannula from 6 mm to 1.5 mm increased the aspirated volume in a near-linear fashion. Extraction sleeves and the luer-trocar system showed significantly greater volumes aspirated in 5 min when compared to polyimide cannulas and even aspirated a comparable volume at 27G. No significant difference in removal speed was apparent between the extraction sleeve and the luer-trocar system

The volume aspirated in 5 min for all tested silicone oils and removal techniques is depicted in Fig. 2.

All commercially available silicone oils could be aspirated with all tested removal techniques. The monitored pressure of the applied vacuum reached its maximum of 600 mmHg roughly 10–15 s after the start of the removal process. For all silicone oils, the shortening of the cannula significantly increased the volume aspirated in 5 min in a near-linear fashion (r^2^ = 0.980, 0.994, 0.971, 0.946, 0.958 and 0.993 for Siluron 1000, Siluron 2000, Siluron Xtra, Siluron 5000, Densiron 68 and Densiron Xtra, respectively). Comparing the shortest to the longest 23G-polyimide cannula, a mean increase in removal speed of 1.57 ml/min, 0.90 ml/min, 0.7 ml/min, 0.63 ml/min, 0.87 ml/min and 2.73 ml/min was observed for Siluron 1000, Siluron 2000, Siluron Xtra, Siluron 5000, Densiron 68 and Densiron Xtra, respectively.

### Removal speed of the extraction sleeve and the experimental luer-trocar setup

The use of the extraction sleeve and the luer-trocar system majorly increased the aspirated volume of silicone oil. Depending on the tested oil, the removed volume was up to fourfold increased (Siluron 5000) when using a 23G-trocar compared to the shortest (1.5 mm) 23G-cannula. The mean increase in removal speed was 13 ml/min for Siluron 1000, 9.1 ml/ min for Siluron 2000, 3.6 ml/min for Siluron Xtra, 3.1 ml/min for Siluron 5000, 9.1 ml/min for Densiron 68 and 13.8 ml/min for Densiron Xtra, respectively. (p < 0.001 for all oils, unpaired t-tests, see Fig. 2). At 25G, the extraction sleeve and the luer-trocar still outperformed the 23G-cannula significantly by aspirating twice the volume. (p < 0.001, unpaired t-test, see Fig. 2) Even at 27G, the luer-trocar and extraction sleeve performed similarly to the shorted polyimide cannula at 23G allowing the time efficient extraction of silicone oil at a small gauge. For all tested silicone oils, the removal speed of the luer-trocar system was not statistically different compared to the extraction sleeve. (p > 0.90 for all oils).

### Feasibility experiments in vitrectomized porcine eyes

**Fig. 3:**
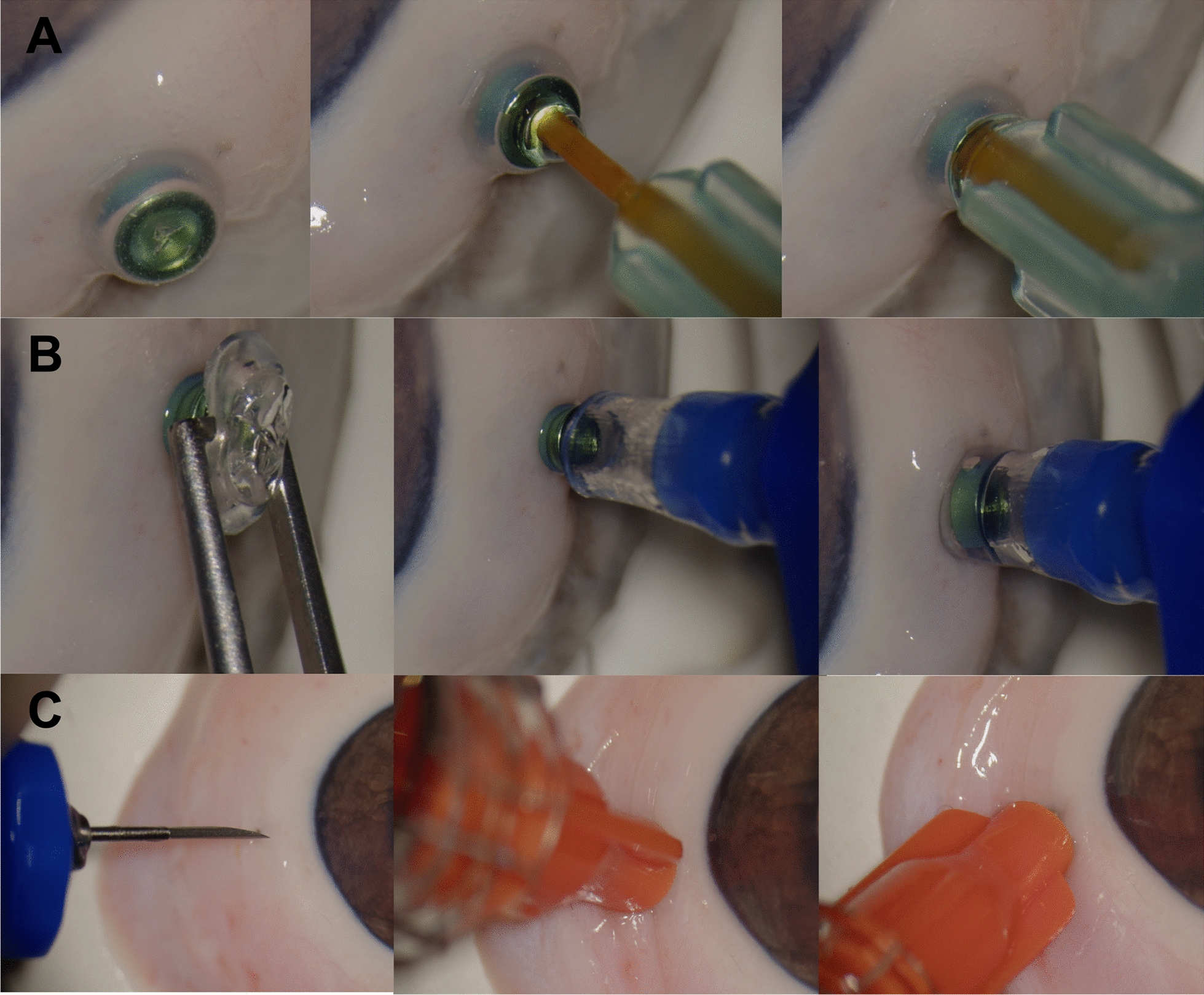
Feasibility experiments on porcine eyes** a**: Unproblematic insertion of a 3 mm 23G-polyimide cannula. The angulation of the trocar to place the trocar tip in the remaining oil bubble was easily achievable. **b**: Placement of the extraction sleeve over a 23G-trocar. In comparison to the shortest cannula, a fourfold increase of the volume aspirated in 5 ml was observed. Angulation of the trocar is not possible. Surgeons must be careful to not lose contact to the trocar during removal. **c**: The luer-trocar system allowed easy angulation of the trocar tip within the eye. The luer-trocar system had similar removal speeds as the extraction sleeve but offered preferable stability and surgical control

During silicone oil removal surgery, it may be necessary to angulate the trocar or the eye using the inserted cannula to aspirate the remaining silicone oil bubble. Thus, we evaluated all polyimide-cannulas (6 mm, 4 mm, 3 mm, 2 mm, 1,5 mm), the extraction sleeve and the luer-trocar system in a porcine eye feasibility study. Angulating the trocar was not impaired for cannula lengths of 3, 4 and 6 mm. While smaller movements without the dislocation of cannula tip were still possible with a length of 2 mm, a length of 1.5 mm proved to come along with reduced control. Similarly, the extraction sleeve did not allow the positioning of the trocar aperture. As silicone oil may act as a lubricant, caution must be taken when using the extraction sleeve to not lose contact with the trocar. The luer-trocar system was stable and allowed the angulation of the trocar with ease. Figure 3 depicts the application of the 23G-polyimide cannula (3 mm), the extraction sleeve and the luer-trocar system.

## Discussion

In this study, we evaluated three techniques to remove silicone oil from the vitreous cavity. A steady increase of flow was observed when shortening the cannula from 6 mm to 1.5 mm, the use of an extraction sleeve or a luer-trocar system massively decreased the time to explant a variety of silicone oils. Although all solutions significantly decreased removal time, there may be some surgical drawbacks due to a decreased ability to angulate the corresponding trocar. This was apparent when using the 1.5 mm 23G-polyimide cannula as well as the extraction sleeve. The third technique, a luer-trocar system that allows a direct connection of the trocar with any medical syringe, combined the advantages of both techniques.

To this date, few laboratory research has been conducted to optimize surgical gear when using high-viscosity silicone oils. Williams et al. in [9] made an effort to improve the injectability and removal of silicone oil by using the effect of “shear thinning”. In fact, the present study includes 3 silicone oils, namely Siluron 2000, Siluron Xtra and Densiron Xtra, that make use of this approach. By creating a high-viscosity silicone oil by mixing low-viscosity silicone oil with high molecular weight components instead of using a one component silicone oil with a medium chain length, favorable effects of the injectability can be achieved while still showing comparable or even better results in the tendency to emulsify [5, 13]. In this study, we focused on improving the injection and removal of silicone oil from the vitreous cavity by shortening the improved cannula made from a rigid, high-performance polymer named polyimide [10]. Without any change to the standard operating procedures of a silicone oil removal surgery we saw an increase in flow of up to 158% (Siluron 5000) when using a shorter cannula. While already a substantial amount of time can be saved by using a polyimide-cannula instead of conventional metal cannulas [10], the shortening decreases the removal time in a scenario of 5 ml of Siluron 5000 by an additional 8 min.

Further, we tested the use of extraction sleeves. While some authors have suggested its use [12], to our knowledge, its performance have never been systematically compared to other silicone oil removal techniques in a controlled laboratory setting. While the increase in flow must be acknowledged, the extraction sleeve also holds three weaknesses that became apparent during the feasibility study. 1. It does not allow the angulation of the trocar tip. 2. The connection between the sleeve and the trocar is susceptible to disconnection through small movements. 3. The extraction sleeve can only be used during the removal process and not during silicone oil injection.

The third technique evaluated in this study is a prototype model of a luer-trocar. The luer-trocar offered optimal conditions for silicone oil removal and may offer surgical innovations not limited to silicone oil surgery in the future. A tight connection to a tube or syringe may improve other current solutions in vitreoretinal surgery. A possible application could be the infusion flow. Indeed, while a luer-trocar system is not commercially available, the recently developed EVA Aveta trocar system by DORC features a Push-Fit connection that shows similar traits to the luer-trocar system studied here and is currently used for fluid infusion during surgery. With small adjustments, specifically a connecting piece between the push-fit trocar and a luer-syringe, the system could enable a luer-trocar system for silicone oil removal to be quickly implemented in clinical practice.

In the past, multiple clinical studies dealt with the removal of silicone oils. Ohayon et al. [14] presented an inexpensive two-trocar setup. However, the oil type used is not mentioned in the article and the exact setup of the active removal trocar is not well described, as the focus of the study is the safety of a two-trocar approach. Priester et al. compared two-trocar approaches in a clinical study. They used a hybrid approach with one 23G-port and one 25G-port compared to a standard 23G-approach. Again, in the article, the used cannula or system for the active removal trocar is not mentioned. As such, again no real comparison of results is possible [15]. Another article on silicone removal was published by Patwardhan et al. in [16]. Goal of this study was to compare a passive, sutured 20G-approach with a passive 23G sutureless approach. As our study uses active removal and focuses on the usage of the trocar rather than their gauge itself, our study is able to expand on this knowledge. Similarly, Jusufbegovic in [17] showed that even a 25G sutureless approach is more time efficient compared to a 20G approach requiring sutures. Again, the optimal usage of the extraction trocar was not the main study question. Song et al. [18] introduced an early version of an extraction sleeve. For that, they modified a blood transfusion system while using a 23G- setup with 3 trocars. Similarly, to the extraction sleeve in this study, the valve has to be removed. However, this approach was not compared to other techniques. Zhang et al. in [19] presented a similar extraction sleeve technique with a 27G setup. However, they did not use an automatically controllable approach to generate vacuum but used a 10 ml syringe in which the plunger is lifted by surgeon to generate vacuum. The approach requires both hands of the surgeon to be involved and vacuum generated by this approach was not quantified appropriately. Finally, the only systematic approach comparing a cannula and an extraction sleeve under controlled conditions was presented by Tabandeh et al. at ARVO 2014. However, the group focused on low-viscosity silicone oil with 1000 mPas. The only study presenting a similar approach to our experimental luer-trocar was published by Akkan et al. [20]. The authors present a common 19G-extraction cannula with a thickened tip that allows the occlusion of a 23G-trocar. In the luer-trocar presented here, a direct connection between the luer-setup and the trocar is provided, the 19G-extraction cannula is interposed in the setup by Akkan et al. [20]. Both approaches provide benefits and should be further evaluated in a comparison study.

In the meanwhile, possible hybrid approaches that first use an extraction sleeve followed by an approach with a cannula to remove the residual silicone oil may be a good option in clinical practice for high-viscosity silicone oils. Due to the greater time savings when explanting high-viscosity silicone oils, possible delays caused by exchanging the sleeve for the cannula may be tolerable.

In conclusion, high-viscosity silicone oil removal can be expedited using shortened polyimide-cannulas, extraction sleeves as well as luer-trocars in the near future. These time savings may translate to shorter surgery times for patients and are easy to implement in daily clinical practice.

## Data Availability

Data is available from the corresponding author upon reasonable request.
